# Efficacy of LNP@2DG-DON liposomal nanoparticles in tumor inhibition and immune activation

**DOI:** 10.1186/s12967-026-08039-8

**Published:** 2026-05-05

**Authors:** Jianang Li, Jiale Feng, Runjie Liu, Yueshan Du, Yueheng Li, Liang Liu, Lei Zhang

**Affiliations:** 1 Department of Pancreatic Surgery, Zhongshan Hospital, Fudan University, 180 Fenglin Road, Shanghai, 200032 People’s Republic of China; 2 Cancer Center, Zhongshan Hospital, Fudan University, Shanghai, 200032 China; 3 Department of General Surgery, Zhongshan Hospital, Fudan University, Shanghai, 200032 China; 4 Department of Basic Medicine, Shanxi Medical University, Taiyuan, Shanxi 030000 China

**Keywords:** 2-deoxy-D-glucose, DON, Liposomal nanoparticles, Metabolic therapy, Immunotherapy, PD-L1, GFAT1, Pancreatic cancer

## Abstract

**Background:**

Pancreatic cancer has poor prognosis, and immunotherapy efficacy is limited by PD-L1 stability via GFAT1-mediated glycosylation. We developed LNP@2DG-DON, a liposomal nanoparticle co-delivering glycolysis inhibitor 2DG and GFAT1 inhibitor DON, to enhance antitumor immunity and metabolic targeting.

**Methods:**

LNP@2DG-DON nanoparticles were synthesized using the thin-film dispersion method and characterized for size, stability, drug loading, and release profiles. In vitro studies assessed cytotoxicity, apoptosis, migration, and invasion in ASPC-1 and PANC-1 pancreatic cancer cell lines. In vivo efficacy was evaluated in a subcutaneous xenograft mouse model, measuring tumor growth, immune cell infiltration, and survival. Western blotting, flow cytometry, and immunohistochemistry were employed to analyze molecular and cellular mechanisms.

**Results:**

LNP@2DG-DON showed uniform size (100–120 nm), high drug loading (6.84–9.27%), and sustained release. It significantly reduced cell viability, induced apoptosis, and inhibited metastasis in vitro. In vivo, it suppressed tumor growth, prolonged survival, and increased CD8+/NK cell infiltration while reducing immunosuppressive cells. Mechanistically, it downregulated PD-L1/GFAT1 and activated pro-apoptotic pathways.

**Conclusions:**

The dual-targeting LNP@2DG-DON nanoparticle synergistically combines metabolic and immune modulation, demonstrating superior antitumor effects compared to single-agent therapies. This approach represents a promising strategy for pancreatic cancer treatment, warranting further clinical investigation.

**Supplementary Information:**

The online version contains supplementary material available at 10.1186/s12967-026-08039-8.

## Introduction

 Pancreatic cancer is still a common and deadly form of cancer in the whole world [1]. Curative treatment has advanced, but mitigating procedures for late-stage pancreatic cancer still record limited success rates [2–5], calling for stronger protocols. Immunotherapy refers to the modern types of treating cancer, which give patients with cancer new hopes of surviving for a long [6–9]. Immune checkpoint inhibitors, such as PD-L1 antibodies, have been a significant breakthrough in the fight, especially against pancreatic cancer [10–12]. PD-L1 protein stability is dependent on GFAT1, which is the rate-limiting enzyme in the hexosamine biosynthetic pathway. GFAT1 produces UDP-N-acetyl-β-glucosamine, which is required for glycosylation. Inhibition of GFAT1 leads to PD-L1 deglycosylation and accelerated proteasomal degradation [13]. Evidently, GFAT1 is required for PD-L1 protein stability, while its blockade lowers PD-L1 levels and stability, thereby promoting T cell and NK cell activation that could enhance responsiveness to immunotherapy in lung cancer [13]. It is thus necessary to develop GFAT1-based approaches in order to block PD-L1.

2-Deoxy-D-glucose (2-DG) is a glucose analog that acts as a competitive glycolytic inhibitor within cells by interfering with D-glucose metabolism and, consequently, ATP production [14–17]. This suggests it could be used as a potential treatment for cancer, as it has been shown to inhibit growth, leading to the death of tumor cells [14–17]. This is currently being studied in various combination settings of clinical chemotherapeutic agents against different cancers [14–17].

Recent studies show that abnormal glycoprotein N-glycosylation levels are critical for pancreatic tumor development [18–22]. Furthermore, pancreatic ductal adenocarcinoma has been found to exhibit changes in N-glycan quantity through quantitative glycoproteomics analysis [18, 22]. They protect the tumor from destruction by CAR T cells by disrupting appropriate immune synapse formation and reducing transcriptional activation, cytokine production, and cytotoxicity [23]. On the PD-1/PD-L1 axis, N-glycans make PD-L1 a formidable obstacle while enhancing CAR T cell activity by maligning the tumor [23]. Other reports suggest that 2DG increases GFAT1 phosphorylation, thereby causing death via endoplasmic reticulum stress and interference with protein N-glycosylation in pancreatic cancers [24]. Combination therapy targeting glycolysis alongside PD-L1 glycosylation might prove a beneficial treatment approach for pancreatic cancer.

Polypharmacology, which uses multiple proteins, has become a promising treatment to counter the shortcomings of single-target therapies. Multi-target therapies have been demonstrated to enhance progression-free survival and efficacy in cancer treatment in clinical trials, including PAPILLON (NCT04538664) and KRYSTAL-1 (NCT03785249) [25, 26]. These treatments minimize side effects by enabling lower drug doses and targeting multiple pathways. The promiscuity of a drug, its ability to engage with multiple targets, is important for increasing therapeutic efficacy [27]. Recent studies point to the growing role of multi-target molecular therapies in addressing cancer resistance and heterogeneity. Nanomaterials made of mesoporous silica have demonstrated potential for co-delivery of multiple therapeutic agents, enhancing the efficacy of combination therapies by targeting multiple pathways simultaneously [27, 28]. These versatile nanomaterials can enhance the performance of cancer therapy by providing a multifaceted treatment strategy. Liposome nanoparticles are among the most important functional platforms for modern cancer therapy, having reached clinical reality [29–32]. These types of nanoparticles can be loaded with anticancer drugs, such as chemotherapy agents, small-molecule inhibitors, nucleic acids, and immunotherapeutic agents [30–34]. Owing to the encapsulation of such agents within liposomes, several advantages can be achieved in cancer treatment. Liposomal nanoparticles alter the pharmacokinetics and biodistribution of loaded drugs, resulting in sustained release and greater accumulation at tumor sites, minimizing off-target effects and systemic toxicity [35, 36]. Furthermore, the outer layer of liposomes can be engineered to carry target ligands or antibodies that bind only to cancer cells, thereby enhancing drug accumulation and boosting curative effects [36]. Creating liposomes that deliver two therapeutic agents simultaneously is an active area of research to enhance cancer immunotherapy by increasing agent levels at the tumor site, leveraging potential synergistic effects, and overcoming drug resistance more effectively than single-agent treatments [37, 38]. Thus, we hypothesized that loading 2DG into the outer layer and DON activators into the liposomes could achieve maximum therapeutic impact against cancer cells, ensuring more targeted and efficient delivery of these important agents and revolutionizing tumor immunotherapy.

In this study, we present an innovative liposomal system designed to enhance the synergistic efficacy of immune checkpoint blockade (ICB) and metabolic therapy in pancreatic cancer. The external layer of the liposome was specifically designed to carry DON, 2DG, or both. DON, a GFAT1 inhibitor, and 2DG, an inhibitor of ATP. By inhibiting Treg cells and promoting PD-L1 degradation, 2DG enhances the efficacy of DON and strengthens immune therapy against pancreatic cancer. The integration of these two distinct therapeutic strategies into a single delivery system is expected to yield a more comprehensive and potent antitumor response. We established both in vitro and in vivo cancer models to evaluate the efficacy of a new liposomal nanoparticle, LNP@2DG-DON, for tumor treatment. Our findings show that LNP@2DG-DON significantly reduces tumor cell viability, increases immune cell infiltration, and activates key immune pathways. In our animal models, the treatment resulted in a significant reduction in tumor size and weight, as well as a longer lifespan. We conclude that the therapeutic benefits of Liposomal LNP@2DG-DON nanoparticles greatly exceed those of 2DG or GFAT1 inhibitors when used alone. In this context, our LNP@2DG-DON system, which combines 2DG and DON, targets both tumor metabolism and immune modulation. This multi-target therapy disrupts PD-L1 glycosylation, enhancing anti-tumor immunity and offering a novel strategy for cancer treatment.

## Materials and methods

### Reagents and cell lines

Various consumables and instruments were used during the experiment. The consumables used included reagents such as DSPE-MPEG (Avitu, Catalog number 2000 147867-65-0), DSPC (Avitu, CAS number 816-94-4, Catalog number S01005), cholesterol (Avitu, CAS number 57-88-5, Catalog number O01001), 6-Diazo-5-oxo-L-nor-Leucine (MCE, CAS number 157-03-9, Catalog number HY-108357), 2-Deoxy-D-glucose (MCE, CAS number 154-17-6, Catalog number HY-13966), absolute ethanol (Shanghai test, Catalog number 64-17-5 100092680), and PBS (Biyuntian, Catalog number C0221A). Additionally, various instruments were used, including an analytical balance (Sartorius, Model BSA124S-CV), ultrasonic cleaner (Kunshan Shumei, Model KQ5200DA), magnetic stirrer (IKA, Model RCT basic), particle size analyzer (Malvern, Model Zetasizer Nano ZS90), refrigerated centrifuge (Lu Xiangyi, Model 3-18R), liposome extruder (Avanti, Catalog number 610000-1EA), and microplate reader (Thermo Fisher Scientific, Model Multiskan MK3).

The ASPC-1 and PANC-1 cell lines were procured from the Shanghai Cell Bank of the Chinese Academy of Sciences (Shanghai, China). Both cell lines were cultured in DMEM (Dulbecco’s modified Eagle’s medium) supplemented with 10% FBS (fetal bovine serum) and 1% penicillin-streptomycin solution. The cells were maintained in a humidified incubator at 37 °C with 5% CO2.

### Preparation of nanoparticles

The experimental protocol involved dissolving DSPC, cholesterol, and DSPE-MPEG2000 in an ethanol solution, and adding the 2DG/DON solution drop by drop to the mixture under continuous stirring. The oil phase was prepared by weighing DSPC (79 mg), cholesterol (19.34 mg), and DSPE-MPEG-2000 (20 mg), respectively, dissolving them in 1 mL of ethanol, and heating to dissolve at 50 °C. A 2DG solution was prepared by adding 10 mg of 2DG to 5 mL of pure water, ultrasonicating for 10 min, and preparing a 2 mg/mL 2DG aqueous solution for later use. Similarly, a DON solution was prepared by adding 10 mg of DON to 5 mL of pure water, ultrasonicating for 10 min, and preparing a 2 mg/mL DON aqueous solution for later use. The LNP was prepared by slowly adding 100 µL of oil phase into 1 mL of ultrapure water solution through a syringe, stirring at 500 r/min for 30 min, leaving it at room temperature for 20 min, stirring at 45 °C for 30 min, and then using a liposome extruder to extrude the liposomes. Co-extrusion was performed to obtain a stable liposome solution with uniform particle size. The LNP@2DG/LNP@DON and LNP@2DG-DON were prepared by slowly adding 100µL oil phase through a syringe into 1mL 1 mg/mL 2DG/DON solution and 1 mL of a solution containing 1 mg/mL 2DG and DON, respectively, stirring at 500 r/min for 30 min, leaving it at room temperature for 20 min, stirring again at 45 °C for 30 min, and then passing through the liposome extruder. The liposomes were co-extruded to obtain a stable liposome solution with uniform particle size. Finally, a 10kd ultrafiltration tube was used to perform ultrafiltration to obtain the samples LNP@2DG and LNP@2DG-DON. The encapsulation rate and loading rate were determined according to the difference method. Briefly, the loading rate and encapsulation rate were calculated by diluting the solution under ultrafiltration appropriately, using a microplate reader to test the absorbance of the solution, substituting it into the standard curve to calculate the mass of the drug in the filtrate, and subtracting the mass of the filtrate from the total dosage to get the mass of the loaded drug. The concentration of DON was calculated by LC-MS, and the encapsulation efficiency and loading rate were calculated accordingly.

### Characterization of nanoparticles

In the particle size and potential test, LNP, LNP@2DG, LNP@DON, and LNP@2DG-DON were each diluted with ultrapure water and subjected to particle size and potential analysis using the Zetasizer Nano ZS90 particle sizer.

The morphology of the nanoparticles was then observed using a Thermo Fisher Talos L120C, and the changes in functional groups in LNP, LNP@DON, LNP@2DG, and LNP@2DG-DON were observed using a Thermo Fisher Nicolet is5 Fourier transform infrared spectrometer, proving the successful loading of 2DG and DON. In order to evaluate the drug release behavior of LNP@2DG, LNP@DON, and LNP@2DG-DON nanodrug-carrying systems, in vitro drug release experiments were conducted. Three parallel drug-loading systems were sealed in dialysis bags, placed in sample bottles containing 10 mL of pH 7.4 buffer, and continuously shaken at 37 °C and 150 r/min for 6, 8, 10, 12, and 24 h. One milliliter of the solution was taken at each time point and replaced with 1 mL of corresponding PBS. LC-MS was used to measure the contents of DON, and the amount of release was calculated based on the standard curve for different time points. The indirect spectrophotometry method was utilized to measure 2DG on the surface of nanoparticles, and their absorbances were recorded using a Multiskan GO microplate reader by Thermo. This indirect method was also used to measure the levels of DON and 2DG in the solution, and the release amount was calculated based on the standard curve at different time points [39].

### Cell culture and treatments

The cells were propagated in DMEM medium with supplementation with 10% FBS and 1% penicillin/streptomycin. The cells were cultured in an incubator with 5% CO2 and temperature set at 37 °C. The cells were pretreated with LNP, LNP@2DG, and LNP@2DG-DON.

### CCK-8 assay for cell viability testing

The cell counting kit-8 (CCK-8) kit (Qayee Bio-Technology) was employed to detect cell viability after the treatment of various nanoparticles. Ninety-six well plates were used to culture the cells. Following that, the cells were placed in the medium for three consecutive days of incubation. We then proceeded to add 10 µl of CCK-8 reagent. After the addition of the reagent, the mixture was left to further incubate for four hours at 37 °C. The microplate reader was used to measure OD values at 450 nm.

### Detection of live/dead cell ratio

Detection of live/dead cell ratio of ASPC-1 and PANC-1 cells treated with various nanodrugs was carried out with the Calcein-AM/PI DoubleStain Kit according to the provided manuel. The cells were cultured for 3 days and then examined using a confocal laser scanning microscope (CLSM, FV1000, Olympus, Japan).

### Immunofluorescence microscopy analysis of cellular uptake

ASPC-1 and PANC-1 cells were placed on chamber slides and left to incubate at 37 °C until they reached 50% confluency. FITC-labeled nanoparticles were added to the cells and then incubated at 37 °C. After 0、2、4、6、8 h, the cells were washed with PBS. And then examined using a confocal laser scanning microscope (CLSM, FV1000, Olympus, Japan).

### Flow cytometry analysis of cellular uptake

ASPC-1 and PANC-1 cells were cultivated in culture flasks with a density of 1 × 105 cells/mL till they reached 60–70% confluence. FITC-labeled nanoparticles were added to the cells and then incubated at 37 °C. After 6 h, the cells were washed with PBS, trypsinized, and re-suspended in PBS. The FACSAria Becton Dickinson was used to perform flow cytometry. The median fluorescence intensity of the cell population that had internalized FITC-labeled Cells was determined using the FlowJo software.

### Flow cytometry analysis of cell apoptosis

The cancer ASPC-1 and PANC-1 cells were cultured in an incubator with 5% CO2 at 37 °C, as indicated above. After 48 h of treatment with the nanodrugs, the cells were harvested and stained with both Annexin and PI for 15 min. Flow cytometry analysis was performed using a CytoFLEX flow cytometer. The percentage of apoptotic cells was analyzed using FlowJo software. The assay was performed in triplicate.

### Cell migration and invasion transwell assays

We entered the transwell chamber into a 24-well plate and added 600 µl DMEM (10% FBS) in every well. Serum-free cell suspensions at a volume of 150 µl were placed in the upper chamber. For the cell invasion assay, the upper layer of the chamber was pre-coated with a thin sheet of Matrigel. In regard to the cell migration assay, a comparable experiment was performed, except that Matrigel was not applied. After a 2-day duration, all chambers were removed, and any leftover liquid was discarded. With a cotton swab, the upper layer of the chamber and any remaining cells in Matrigel were gently wiped away. The cells in the lower layer of the chamber were fixed with 90% ethyl alcohol for 30 min after two washes with PBS. Crystal violet (0.1%) was then implemented to stain cells for 30 min. In five random, nonoverlapping fields of view, the cells that invaded or migrated were counted using a microscope.

### Animal study

To establish a subcutaneous tumor xenograft, 1 × 10^6^ ASPC-1 cells were suspended in 100 µl of culture medium with Matrigel and injected into the flank of 8-week-old male C5**7**BL/6J nude mice. Five groups of six mice each were randomly assigned: the PBS, LNP, LNP@2DG, LNP@ DON, and LNP@2DG-DON. Once the tumor size reached about 100 mm^3^, the different drug groups were administered intravenously via the tail vein six times on days 1,4,7,10,14, and 21. 2DG was given at 2.5 mg/kg for each dose. Tumor volume was monitored weekly. Tumor growth was monitored after 28 days, and tumor weight was calculated using the formula length × width × 0.5 mm ^3^. The tumors were weighed and imaged on one paper, and the major organs (liver, spleen, kidney, heart, and lung) were washed with PBS and fixed in 4% paraformaldehyde for further hematoxylin and eosin (H&E) staining. Animal experiments were conducted in compliance with the Care and Use of Laboratory Animals guidelines of our institution.

### H&E staining

We conducted an experiment to stain tissues using Harris hematoxylin and Eosin. The tissues collected were xenografts and major organs such as liver, spleen, kidney, heart, and lungs. These were cleansed with PBS and preserved with 4% paraformaldehyde. The preserved tissues were embedded in paraffin, sliced into 5 μm sections, and then subjected to staining. Finally, an optical microscope was used to examine the samples.

### In vivo TUNEL assay

Tissues were fixed with ethanol overnight at 4 °C, followed by embedding in paraffin and sectioning. The sections were then subjected to permeabilization with 0.1% Triton X-100 for 15 min at room temperature. The TUNEL reaction was performed using the Fluorescence method according to the manufacturer’s instructions. The sections were then counterstained with Eosin and imaged using fluorescence microscopy.

### Immunofluorescence detection of AMPK in the xenograft tissue

Tissues were fixed with 4% paraformaldehyde overnight at 4 °C, followed by embedding in paraffin and sectioning. The sections were then subjected to antigen retrieval and blocking. The sections were then incubated with primary antibody against anti-AMPK at 4 °C overnight, followed by detection using Alexa Fluor 488-conjugated secondary antibody. The sections were mounted using ProLong Diamond Antifade Mountant and imaged using Confocal microscopy. The expression of the protein was quantified by measuring the fluorescence intensity per unit area using ImageJ software.

### Ki67-immunohistochemical staining of the tumor xenograft tissue

Tumor xenograft tissue was fixed in formalin and embedded in paraffin. Sections of anti-Ki67 were cut and subjected to antigen retrieval. The sections were then incubated with primary antibody for 2 h at room temperature, followed by detection using HRP staining. DAPI was used as a counterstain. The stained sections were imaged using Zeiss optical microscope. The expression of the protein was analyzed by measuring the intensity of staining using ImageJ software.

### In vivo and ex vivo orgnan imaging

For in vivo and ex vivo imaging, DiR-loaded liposomes(LNP@2DG-DON) were injected intravenously at a dose of 200 µg DiR/kg to evaluate in vivo biodistribution at the target tissue sites. Imaging was conducted using a Bruker Xtreme small-animal X-ray and fluorescence bioluminescence imaging system. A Tumor-bearing mice were photographed 0, 4, 8, 12 h after injection using the IVI^®^ Spectrum system (Caliper, Hopkinton, MA, USA). Next, the mice were euthanized by cervical dislocation, and their vital organs and tissues (including heart, liver, spleen, lungs, kidneys, and tumors) were excised for cryosection observation.

### Flow cytometry analysis of immune cells in vivo

After the treatment, the immune activity of mice was investigated by collecting samples like tumors, tumor-draining lymph nodes, and spleen. The tumor was cut into small pieces and digested in a DMEM medium containing 2 mg/mL Collagenase IV and 0.2 mg/mL DNase I. The resulting single-cell suspensions were stained with fluorescence-labeled antibodies to identify different markers like CD3, CD4, CD8, CD69, CD11b, Gr-1, CD80, F4/80, and PDL1. The frequency of T cells and MDSC in TDLN and spleen was also determined using flow cytometry.

### Western blot assay

Proteins were obtained via extraction with the RIPA Buffer (Radioimmunoprecipitation Assay Buffer) containing protease inhibitors. Protein concentration was measured using a BCA Protein Assay kit (Pierce; Thermo Fisher Scientific, Inc.) and 5 mg/mL of protein was loaded onto an SDS-PAGE gel. The membrane was blocked using 5% non-fat milk in TBST and subsequently incubated with the primary antibodies against PD-L1, GFAT1, KI67, Cleaved-Caspase 3, Bcl2, BAX, and β-Actin at 4 °C. Following washing with TBST, we incubated the membrane with the secondary antibody anti-rabbit IgG for 1 h at ambient temperature. The visualization of protein bands was performed using chemiluminescence and quantified using ImageJ image analysis software. ACTIN was used as an internal control.

### Flow cytometry analysis of immune cells in splenocytes and tumor tissues

Splenocytes were obtained using a method that had been established before [37]. To start with, anti-CD16/CD32 mAb (5 µg/mL) was introduced to 2 × 10^6^ splenocytes and incubated at ice temperature for 20 min. Following that, these cells were stained with anti-CD3, CD4, CD8α, CD25 + FOXO3+, NK1-1, Gr-1,CD62L, CD44, CD80, and CD11b Abs (1 µg/mL) for half an hour at ice temperature. After that, they were rinsed with 2% FBS, 1 mM EDTA, and 0.1% sodium azide in PBS. Lastly, the labeled cells were examined using Accuri™ (BD Biosciences) and FlowJo Software (Treestar, Inc., San Carlos, CA, USA).

### Statistical analysis

**Fig. 1:**
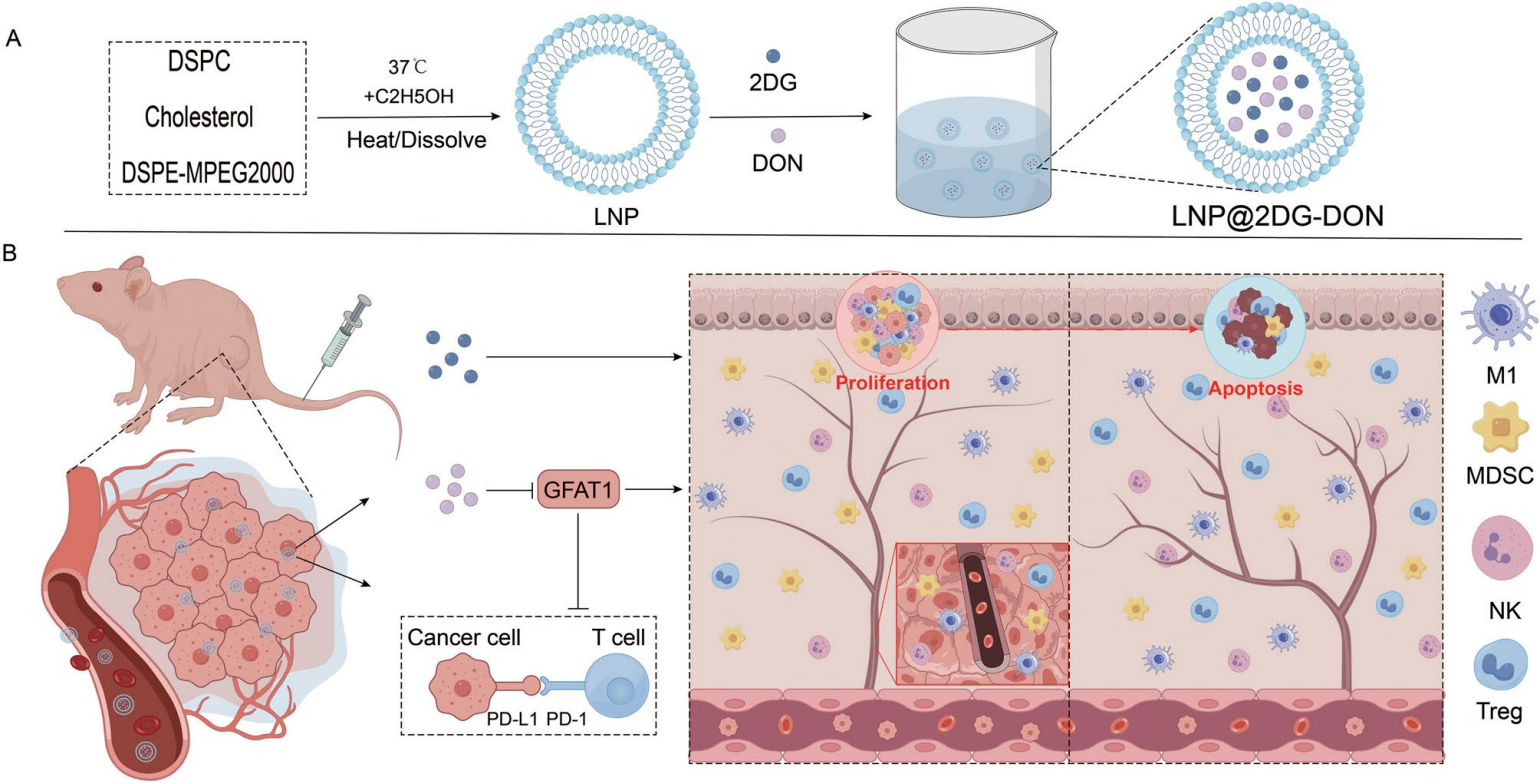
Schematic illustration of LNP@2DG-DON targets tumor metabolic pathways to remodel the immunosuppressive microenvironment and synergizes with immune checkpoint blockade for tumor suppression

The results of the experiment were presented using the mean with standard error of the mean (SEM) and were replicated three or more times. To determine the variations between the groups, a one-way ANOVA was conducted utilizing SPSS22.0(SPSS Inc., USA) and GraphPad Prism 8 (Graph-Pad, San Diego, CA). The statistical significance was indicated by *P < 0.05, **P < 0.01, and ***P < 0.001 (see Fig. 1).

## Results

### Characterization of LNP@2DG-DON

**Fig. 2:**
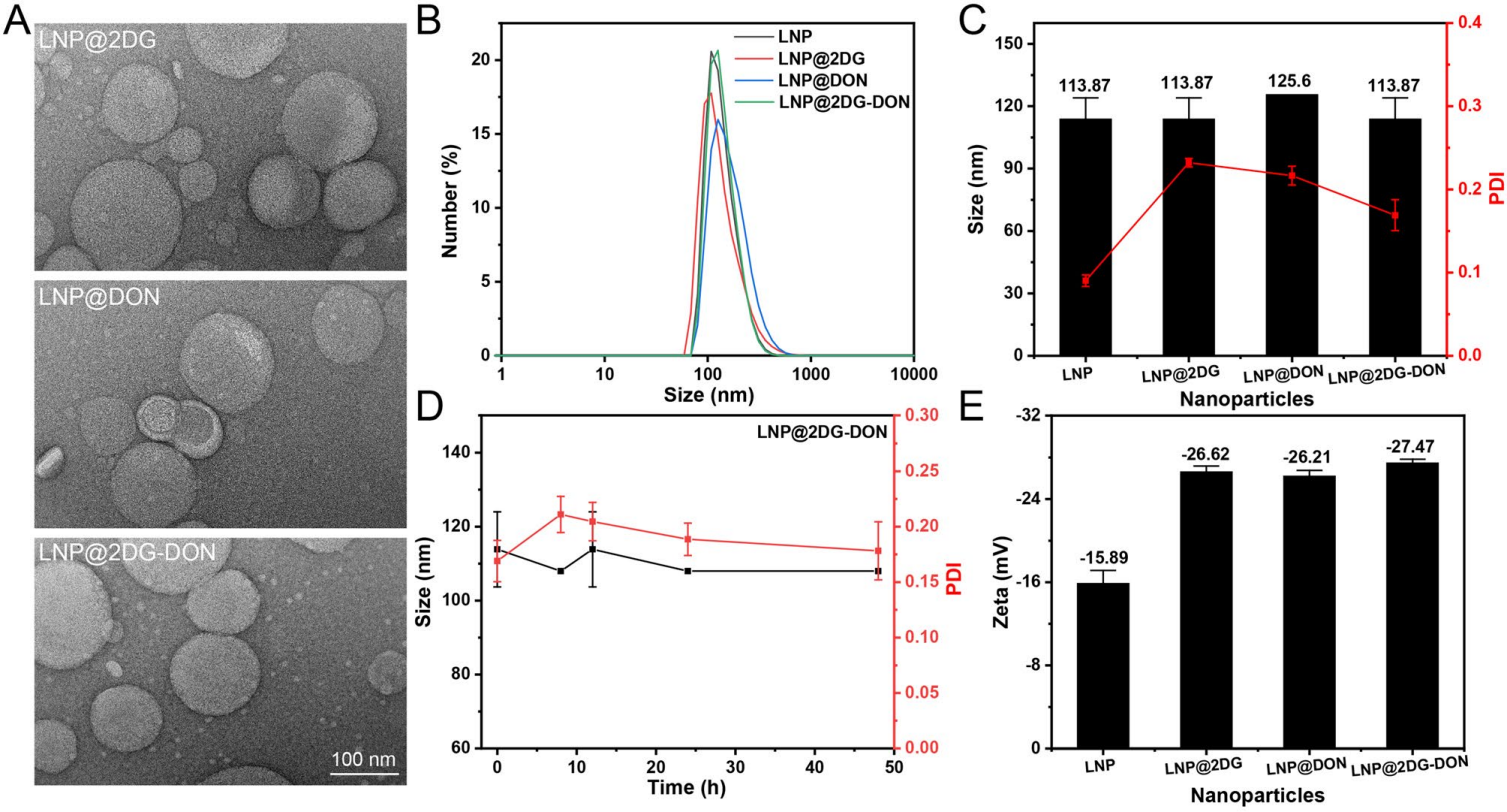
Characterization of LNP. **A**) TEM imaging of LNP@2DG, LNP@DON, and LNP@2DG-DON. **B**) Particle size analysis of LNP@2DG, LNP@DON, and LNP@2DG-DON. **C**) Average particle size and Polydispersity index(PDI) of LNP@2DG, LNP@DON, and LNP@2DG-DON. **D**) Stability analysis of LNP@2DG, LNP@DON, and LNP@2DG-DON. **E**) Zeta potential measurement of LNP@2DG, LNP@DON, and LNP@2DG-DON

**Fig. 3:**
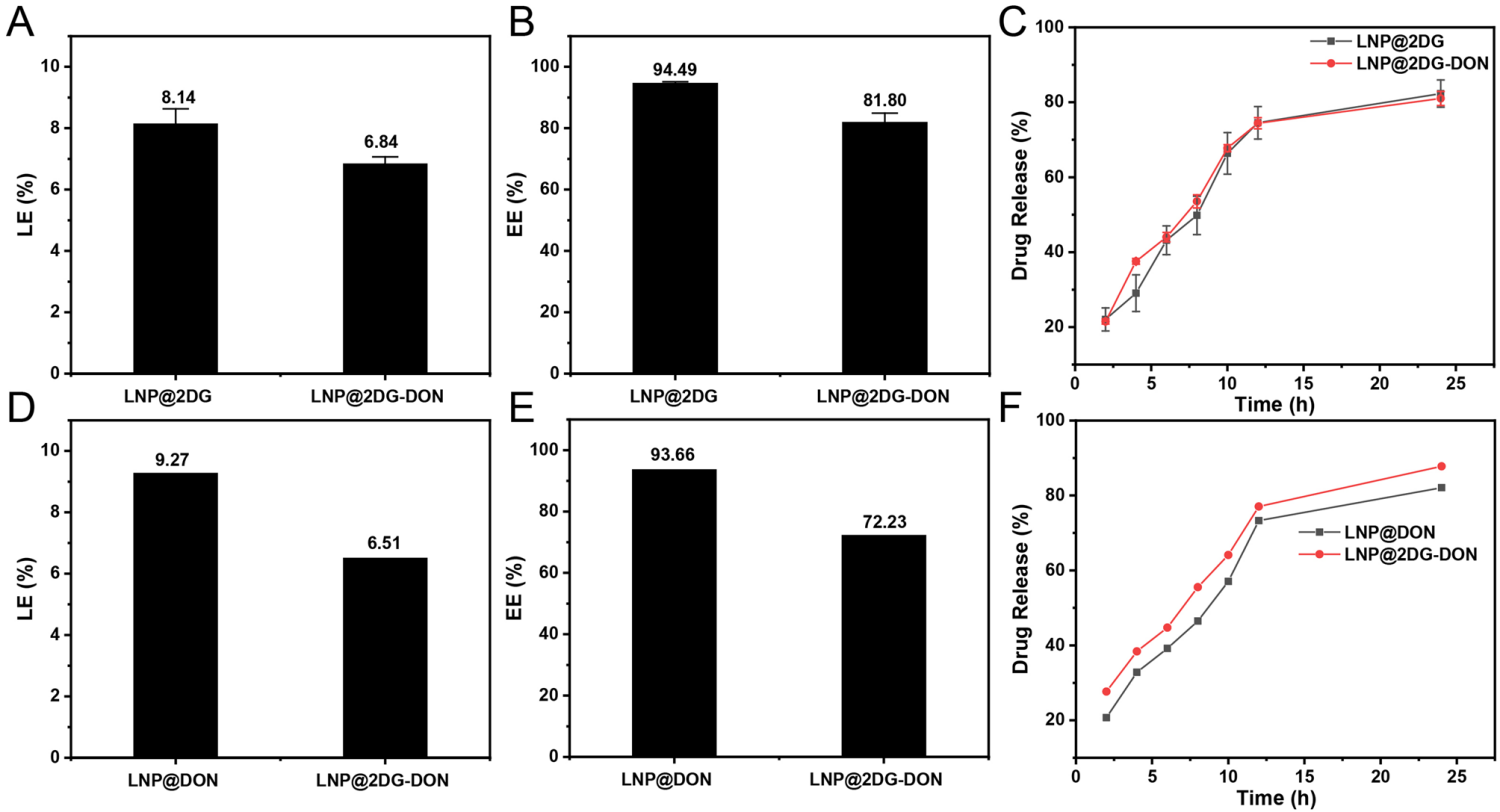
Drug release of LNP@ 2DG and LNP@2DG-DON. **A**) Load efficiency of LNP@2DG and LNP@2DG-DON for 2DGs. **B**) Entrapment efficiency of LNP@2DG and LNP@2DG-DON for 2DGs. **C**) Drug release of LNP@2DG and LNP@2DG-DON for 2DGs. **D**) Load efficiency of LNP@DON and LNP@2DG-DON for DON. **E**) Entrapment efficiency of LNP@DON and LNP@2DG-DON for DON. **F**) Drug release of LNP@2DG and LNP@2DG-DON for DON

In this study, we prepared and characterized 2DG, DON, and LNP-based nano-drugs. Due to its glycosylation status, DON is an inhibitor of GFAT1, a PD-L1 targeting molecule. We used the thin-film dispersion method to successfully prepare the liposome, as seen in Fig. 2. To link the PD-L1 targeting peptide inhibitor (DON) with the liposome, we used the maleimide-thiol reaction. We loaded 2DG into the liposome using the ammonium sulfate gradient method and then diluted and characterized the prepared liposome [40]. TEM microscopy was used to analyze the morphology of LNP-loaded nano-drugs, all of which appeared spherical (Fig. 2A). By dynamic light scattering analysis we found that the majority of the particles were uniformly distributed and had a size range of 80 to 1000 nm for all LNP-types, with a peak around 100 (Fig. 2B and C shows that the hydrodynamic diameters of LNP, LNP@2DG, LNP@DON, and LNP@2DG-DON were 117.53 ± 8.310 nm, while the that of LNP@DON was 122.60 ± 2.646 nm. The Polydispersity Index (PDI) values of LNP (0.09), LNP@2DG (0.24), LNP@DON (0.22), and LNP@2DG-DON (0.18) were within the desired PDI value of 0.3 for pharmaceutical nanoparticle (Fig. 2C). The LNP@2DG-DON showed high stability after 48 h, with the less noticeable changes in its size and PDI (Fig. 2D). The zeta potential of LNP, LNP-loaded nano-drugs were − 15.89, -26.62, -26.21, and − 27.47 mV, respectively (Fig. 2D). The LE (%) for LNP@2DG was 8.14 (Fig. 3A), while that for LNP@2DG-DON was 6.84 (Fig. 3A). The EE (%) values for LNP@2DG and LNP@2DG-DON in ASPC-1 cells were 94.49 and 81.8, respectively (Fig. 3B). The drug release (%) over 24 h for LNP@2DG and LNP@2DG-DON was more than 80% (Fig. 3C). The LE (%) for LNP@DON was 9.27, while that for LNP@2DG-DON was 6.51 (Fig. 3D). The EE (%) values for LNP@DON and LNP@2DG-DON in PANC-1 cells were 93.66 and 72.23, respectively (Fig. 3E). The drug release (%) over 24 h for LNP@DON and LNP@2DG-DON was more than 80% (Fig. 3F). Thus, our results indicated that LNP@2DG-DON has similar characteristics to LNP@DON and LNP@2DG and can be used as a candidate drug delivery system with high stability.

Figure S1 displays the infrared spectra of LNP, LNP@2DG, LNP@DON, and LNP@2DG-DON. LNP exhibited a stretching vibration absorption peak at 3427 cm-1 associated with -OH, absorption peaks near 2919 cm-1 and 2851 cm-1 attributed to symmetric and antisymmetric stretching vibration absorption peaks of CH2 of phospholipid fatty acyl chain, a stretching vibration absorption peak attributed to C = O at around 1736 cm-1, and an absorption peak attributed to the in-plane asymmetric bending vibration of -CH2- and -CH3 at 1468 cm-1. The spectrum of LNP@2DG was similar to that of LNP, with a stretching vibration absorption peak attributed to -OH at 3424 cm-1, a CH2 vibration absorption peak at 2919 cm-1 and 2851 cm-1, and an asymmetric bending vibration absorption peak at 1736 cm-1 and 1468 cm-1 attributed to C = O, -CH2- and -CH3. Moreover, the C-O-C stretching vibration absorption peaks attributed to 2DG were at 1242 cm-1, 1111 cm-1, and 843 cm-1 of LNP@2DG.

### Effect of LNP@2DG-DON on pancreatic cancer cells

**Fig. 4:**
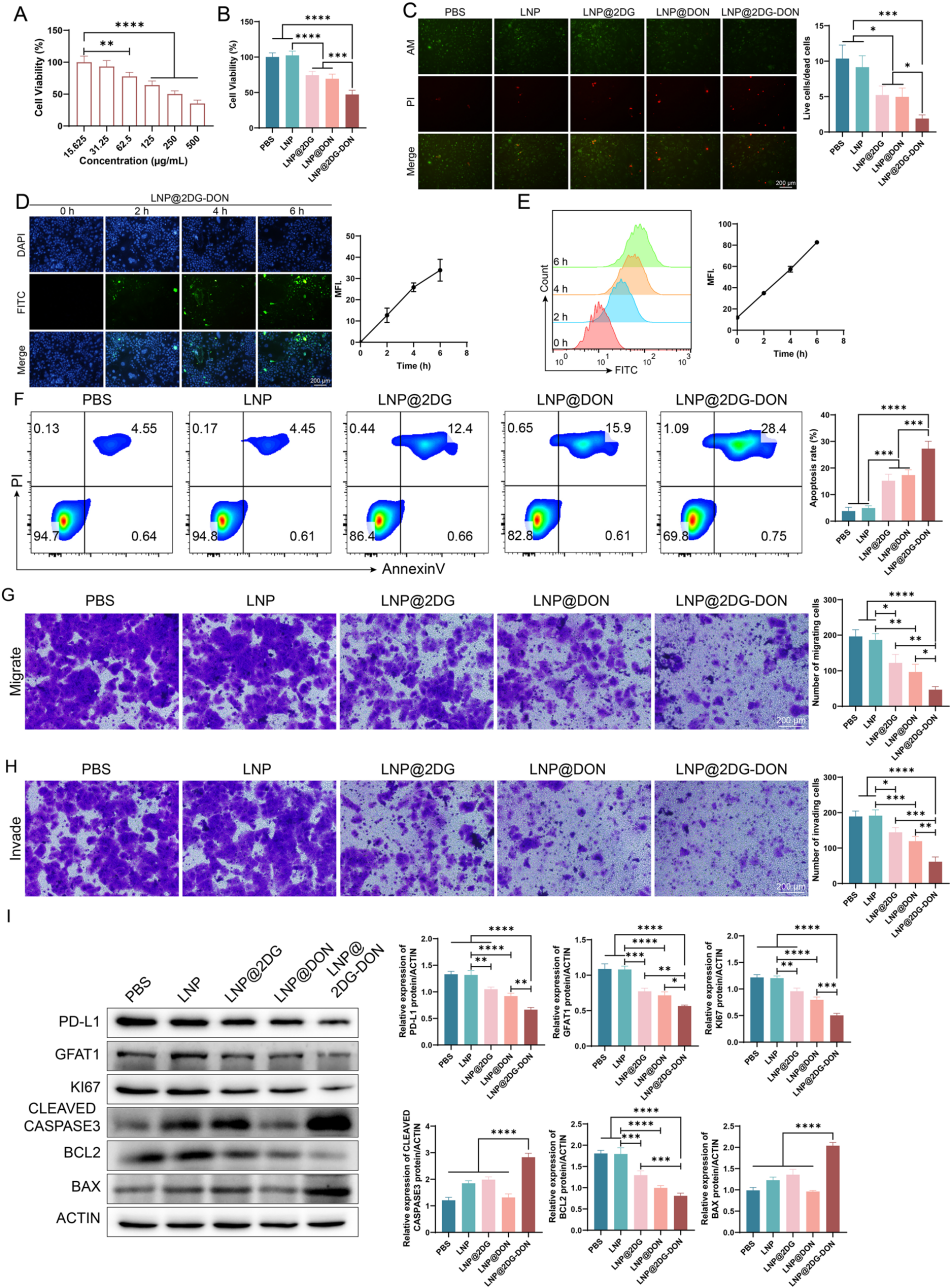
Assessment of cell viability, apoptotic induction, and tumor-fighting in ASPC-1 cells treated with various formulations. **A**) Cell viability percentage of ASPC-1 cells after 48 h of treatment with LNP@2DG-DON. **B**) Comparative cell viability after treatment with PBS, LNP, LNP@2DG, LNP@DON, and LNP@2DG-DON. **C**) Fluorescent microscopy images showing live/dead cells stained with PI (Propidium Iodide, red) and viable cells with Calcein AM (green) across different treatments. **D**) Time-dependent uptake of FITC-labeled LNP@2DG-DON by ASPC-1 cells was observed over 0, 2, 4, 6, 8 h. **E**) Flow cytometry histograms indicating the intensity of FITC signal in ASPC-1 cells post-treatment. **F**) Quantitative analysis of apoptotici cells from flow cytometry data. **G**) Transwell migration and invasion assays and corresponding densitometric quantification after different treatments. **I**) Western blot analysis and corresponding densitometric quantification of PDL-1, GFAT1, KI67, Cleaved Caspase 3, BCL2, and BAX protein levels after treatments, with ACTIN as the loading control. The original, uncropped images of the Western blots are provided in the supplementary materials. Statistical significance is denoted by asterisks with *p < 0.05, **p < 0.01, ***p < 0.001, and ****p < 0.0001, indicating increased efficacy of the LNP@2DG-DON formulation in inducing apoptosis and tumor-fighting compared to other treatments

**Fig. 5:**
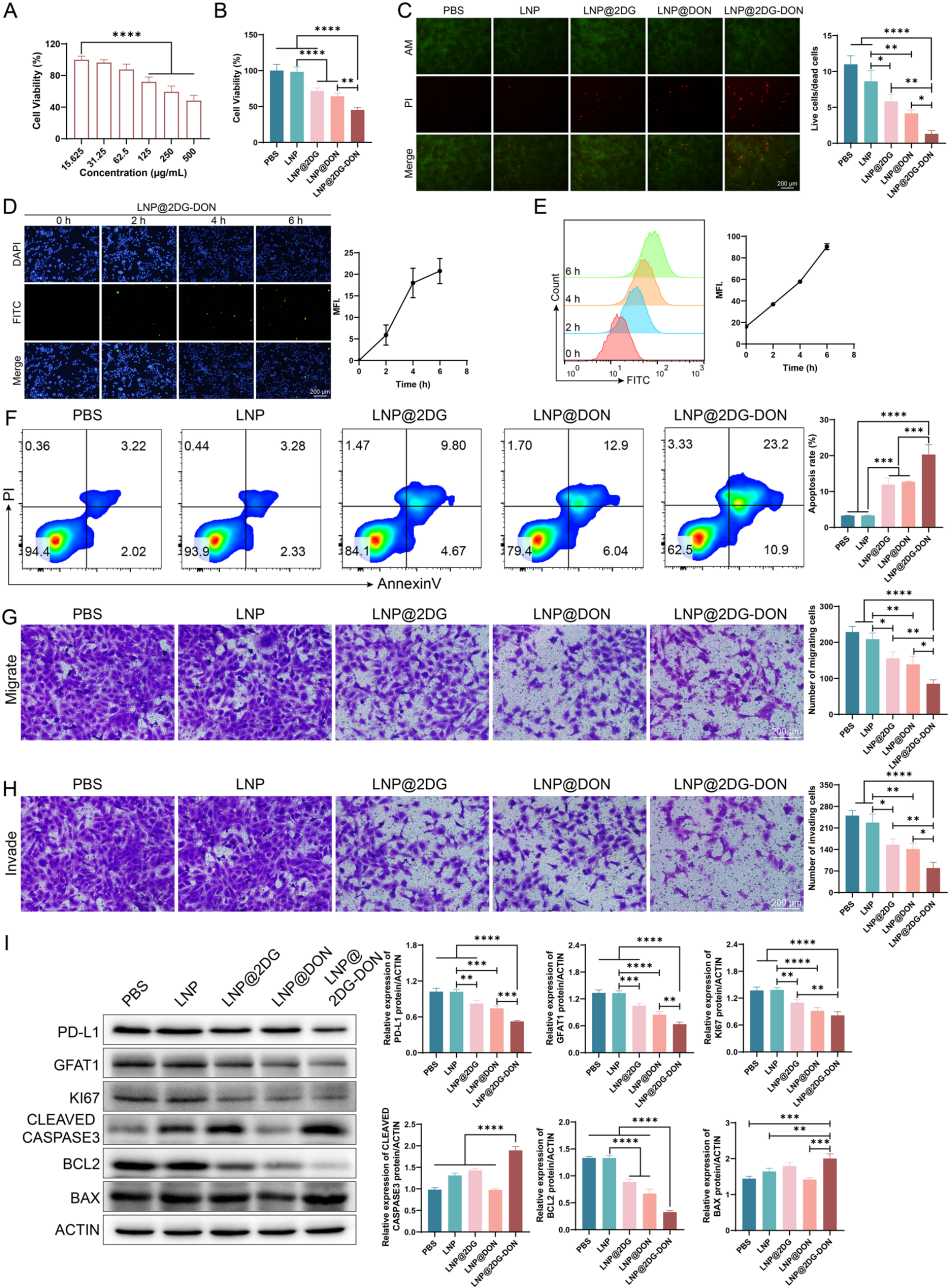
Assessment of cell viability, apoptotic induction, and tumor-fighting in PANC-1 cells treated with various formulations. **A**) Cell viability percentage of PANC-1 cells after 48 h of treatment with LNP@2DG-DON. **B**) Comparative cell viability after treatment with PBS, LNP, LNP@2DG, LNP@DON, and LNP@2DG-DON. **C**) Fluorescent microscopy images showing live/dead cells stained with PI (Propidium Iodide, red) and viable cells with Calcein AM (green) across different treatments. **D**) Time-dependent uptake of FITC-labeled LNP@2DG-DON by PANC-1 cells, observed over 0, 2, 4, 6, 8 h. **E**) Flow cytometry histograms indicating the intensity of FITC signal in PANC-1 cells post-treatment. **F**) Quantitative analysis of apoptotici cells from flow cytometry data. **G**) Transwell migration and invasion assays and corresponding densitometric quantification after different treatments. **I**) Western blot analysis and corresponding densitometric quantification of PDL-1, GFAT1, KI67, Cleaved Caspase 3, BCL2, and BAX protein levels after treatments, with ACTIN as the loading control. The original, uncropped images of the Western blots are provided in the supplementary materials.Statistical significance is denoted by asterisks with *p < 0.05, **p < 0.01, ***p < 0.001, and ****p < 0.0001, indicating increased efficacy of the LNP@2DG-DON formulation in inducing apoptosis and tumor-fighting compared to other treatments

The cytotoxicity effect of the prepared LNP@2DG-DON on pancreatic cancer cells (ASPC-1 cells (Fig. 4A, *p* < 0.05) and PANC-1 cells (Fig. 5A, *p* < 0.05) showed a sharp decline in cell viability in a dose-dependent manner. Next, various assays were performed on pancreatic cancer cells to check the anticancer effect of LNP@2DG-DON on these cells treated with different nano-drugs. The MTT assay showed a reduction in cell viability of ASPC-1 cells (Fig. 4B) and PANC-1 cells (Fig. 5B) treated with LNP-loaded nano-drugs in contrast to LNP and PBS, with statistical significance (*p* < 0.05). Moreover, PI staining showed an insignificant discrepancy in the ratio of live and dead pancreatic cancer cells treated with PBS and LNP. Still, a significant reduction in the ratio of live and dead cells was recorded in cells treated with LNP-loaded nano-drugs in contrast to LNP (Figs. 4C and 5C, *p* < 0.05). The quantification of cellular uptake of FITC-labeled nano-drugs in ASPC-1 cells (Fig. 4D and E, *p* < 0.05) and PANC-1 cells (Fig. 5D and E, *p* < 0.05) were analyzed by immunofluorescence staining or flow cytometry. The FITC immunofluorescence staining of pancreatic cancer cells indicated significantly increased MFI for the groups treated with LNP@2DG-DON time-dependent (Figs. 4D and 5D, *p* < 0.05). Moreover, the MFI for the groups treated with LNP@2DG and LNP@DON also increased significantly at 2 h, 4 h, and 6 h as compared with the control group (0 h) (Fig. S2 and S3, *p* < 0.05). In flow cytometry fluorescence analysis, the MFI change for the cells treated with LNP@2DG-DON was similar to that observed in the immunofluorescence assay (Figs. 4E and 5E, *p* < 0.05), indicating the high cellular uptake of FITC-labeled nanoparticles in both pancreatic cancer cells treated with the nao-drugs.

Moreover, flow cytometry analysis indicated an increase in the apoptosis rate of pancreatic cancer cells treated with LNP-loaded nano-drugs in contrast to LPS- and LNP-treated cells (Figs. 4F and 5F, *p* < 0.05), with a statistically and remarkably increased apoptosis rate in the LNP@2DG-DON group compared to LNP@2DG and LNP@DON (Figs. 4F and 5F, *p* < 0.05). Transwell migration and invasion assays showed a reduction in the migration (Figs. 4G and 5G, *p* < 0.05) and invasion (Figs. 4H and 5H, *p* < 0.05) of pancreatic cancer cells treated with the nano drugs as contrasted to LNP and PBS. Additionally, a significant reduction in the migration (Figs. 4G and 5G, *p* < 0.05) and invasion (Figs. 4H and 5H, *p* < 0.05) of cells treated with LNP@2DG-DON was observed when compared to LNP@2DG and LNP@DON. The results suggest that LNP@2DG-DON significantly affects the apoptosis rate, cell viability, live and dead cell ratio, and migration and invasion of pancreatic cancer cells. The drug combination of LNP@2DG-DON was found to be more effective than LNP@2DG and LNP@DON alone.

The western blot results of proteins in ASPC-1 (Fig. 4I) and PANC-1 (Fig. 5I) cells were compared among the various groups. For BAX expression, insignificant discrepancy was recorded between PBS and LNP groups (Figs. 4I and 5I, *p* > 0.05). However, an increase was recorded in LNP-loaded nano-drugs in contrast to the LNP group. Similarly, for BCL2 and GFAT1 expression levels, a downregulation (*p* < 0.05) was recorded in LNP-loaded nano-drugs in contrast to the LPS AND LNP group. For Cleaved caspase 3, an upregulated expression (*p* < 0.05) was recorded in LNP-loaded nano-drugs compared to the LNP- and PBS-treated cells. For ki67 expression, a reduction was recorded in LNP-loaded nano-drugs in contrast to the LNP and PBS groups. For PD-L1 expression, a reduction was recorded in LNP-loaded nano-drugs in contrast to the LNP and PBS groups. A sharp decline was recorded in LNP@2DG-DON compared to LNP@DON or LNP@2DG (Figs. 4I and 5I, *p* < 0.05).

### Inhibitory effect of LNP@2DG-DON on tumor in vivo

**Fig. 6:**
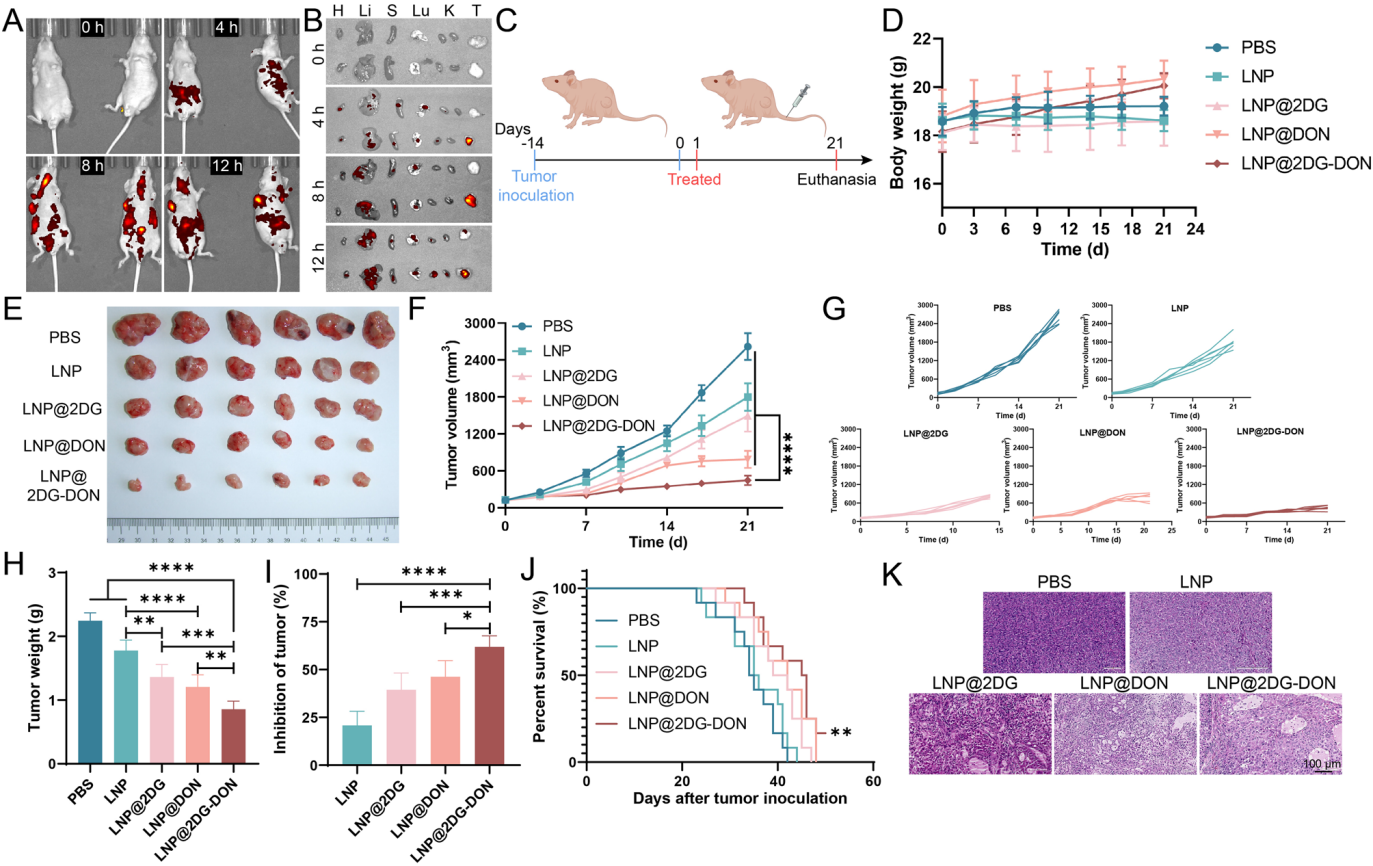
Anti-tumor effects in the subcutaneous pancreatic cancer tumor model. **A**) In vivo biodistribution image after intravenously injecting LNP@2DG-DON 0 h, 4 h, 8 h and 12 h **B**) Ex vivo biodistribution image after intravenously injecting LNP@2DG-DON 0 h, 4 h, 8 h and 12 h in heart (H), liver (Li), spleen (S), lungs (Lu), kidneys (K) and tumor (T). The concentration of (D) **C**) Schematic illustrations of the treatment schedule. **D**) Body weight changes in mice after various treatments. **E**) Collected subcutaneous tumors from different treatments. **F**) Average tumor growth curves for mice bearing pancreatic cancer tumors after various treatments. **G**) Individual tumor growth curves. **H**) Tumor weight changes in mice after various treatments. **I**) Tumor inhibition ratio in different groups. **J**) Percent survival. **K**) H&E staining of subcutaneous tumors

To demonstrate the effectiveness of prepared LNP nano-drugs in treating subcutaneous pancreatic cancer tumors in mice, the anti-tumor efficacy of liposomal drugs in a subcutaneous pancreatic cancer mouse tumor model was assessed across five groups. The in vivo imaging (Fig. 6A) and organ imaging (Fig. 6B) were utilized to confirm successful tumor modeling. The mice were transplanted with cancer cells 14 days before the start of treatment, which commenced on Day 0 (Fig. 6C). A follow-up or additional treatment was given on Day 1 (Fig. 6C). The endpoint of the study was marked by euthanasia on Day 14 to evaluate the effects of the treatment post-mortem (Fig. 6C). The measurement of body weight in mouse with established tumor xenografts showed insignificant changes (*P* > 0.05) in body weight between PBS, LNP, LNP-loaded nano-drugs groups at different time points (Fig. 6D). The effect of various formulations on tumor volume was analyzed at indicated time points (Fig. 6E and G *p* < 0.05). The tumor volume was increased in all groups over 21 days compared to the initial tumor volume (Fig. 6E and G *p* < 0.05). Moreover, in the LNP-loaded nano-drugs groups, the tumor volume was decreased in contrast to the PBS and LNPgroups, and the effect was more pronounced in the LNP@2DG-DON group (Fig. 6E and G *p* < 0.05). In addition, significant differences were observed between the LNP@2DG-DON group and the LNP@2DG or LNP@DON groups (Fig. 6E and G *p* < 0.05). The results also showed that the treatment with LNP-loaded nano-drugs led to a sharp decline in tumor weight compared to the PBS group (Fig. 6H, *P* < 0.05). However, insignificant discrepancies were observed in tumor weight between the LNP and PBS groups (Fig. 6H, *P* > 0.05), while a sharp decline was recorded in the LNP@2DG-DON group in contrast to the LNP@2DG or LNP@DON group (Fig. 6H, *P* < 0.05). Regarding tumor inhibition rate, treatment with LNP-loaded nano-drugs resulted in a significant increase in contrast to the PBS group (Fig. 6I, *P* < 0.05). A sharp decline in LNP@2DG-DON was recorded compared to LNP@2DG or LNP@DON groups (Fig. 6I, *P* < 0.05). Insignificant changes were observed in tumor inhibition rate between the LNP and PBS groups (Fig. 6I, *P* > 0.05). Survival analysis indicated that LNP@2DG-DON considerably prolonged the survival rate of mice with xenografts in contrast to the other groups (Fig. 6J, *P* < 0.05). HE staining also indicated the tumor inhibitory effect of LNP@2DG-DON (Fig. 6K).

### Effects of LNP@2DG-DON on tumor cell proliferation, apoptosis, and protein expression in vivo

**Fig. 7:**
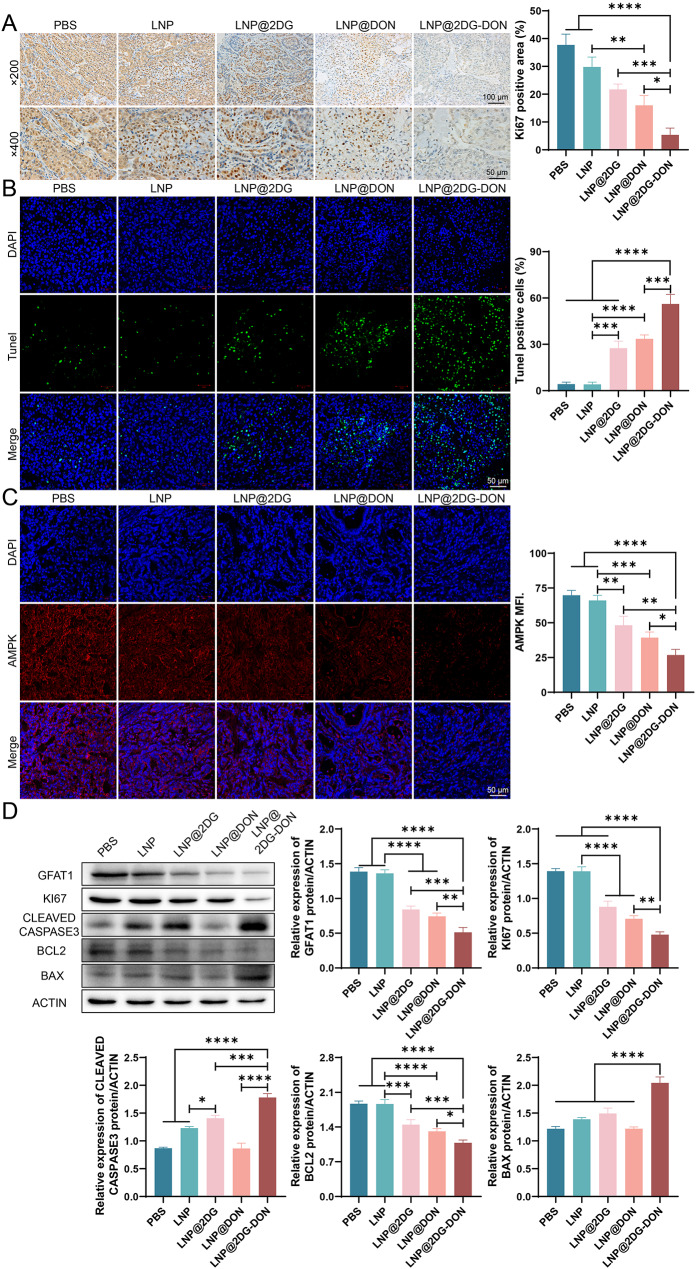
In Vivo Tumor Cell Proliferation, Apoptosis, and Protein Expression changes due to LNP nanoparticles. **A**) Immunohistochemistry and corresponding densitometric quantification of Ki67 in tumors after different treatments. **B**) TUNEL assay in pancreatic cancer tumor tissues. **C**) Immunofluorescence detection of AMPK in pancreatic cancer tumor tissues and quantitative analysis. **D**) Western Blot analysis of the expression levels of GFAT1, KI67, Cleavde Caspase 3, BCL2, and BAX in pancreatic cancer tumor tissues following different treatments. The original, uncropped images of the Western blots are provided in the supplementary materials

To investigate the effect of the LNP nano-drugs on cell proliferation, immunohistochemistry of Ki67 in tumors was performed. The results indicated that the Ki67-positive area in tumor tissues was markedly reduced in the LNP-loaded nano-drugs groups relative to the PBS and LNP groups (Fig. 7A, *P* < 0.05). There was a sharp decline in the ki67-positive area between the LNP@2DG-DON and LNP@2DG or LNP@DON groups (Fig. 7A, *P* < 0.05). The Tunel staining of tumor tissues showed that LNP-loaded nano-drugs markedly increased in Tunel-positive cells compared to the PBS and LNP groups (Fig. 7B, *P* < 0.05). Insignificant discrepancies were observed in Tunel-positive cells between the LNP and PBS groups (*P* > 0.05), while a significant increase was recorded between the LNP@2DG-DON and LNP@2DG or LNP@DON groups (Fig. 7B, *P* < 0.05). Moreover, the immunofluorescence analysis showed that AMPK MFI was markedly reduced in the LNP-loaded nano-drugs groups in contrast to the PBS and LNP groups, and a sharp decline was recorded between the LNP@2DG-DON and the LNP@2DG or LNP@DON groups (Fig. 7C, *P* < 0.05). However, insignificant change was recorded in the AMPK MFI between the LNP and PBS groups (Fig. 7C, *P* > 0.05). Western blotting analysis revealed that treatment with LNP-loaded nano-drugs led to a significant increase in BAX and cleaved caspase3 protein expression levels and a sharp decline in BCL2, ki67, and PD-L1 expression in contrast to the PBS and LNP groups (Fig. 7D, *P* < 0.05). In addition, significant differences were observed in BAX, cleaved caspase3, BCL2, ki67, and PD-L1 expression between the LNP and PBS groups (Fig. 7D, *P* > 0.05).

### Effect of LNP@2DG-DON on immune cells in vivo

**Fig. 8:**
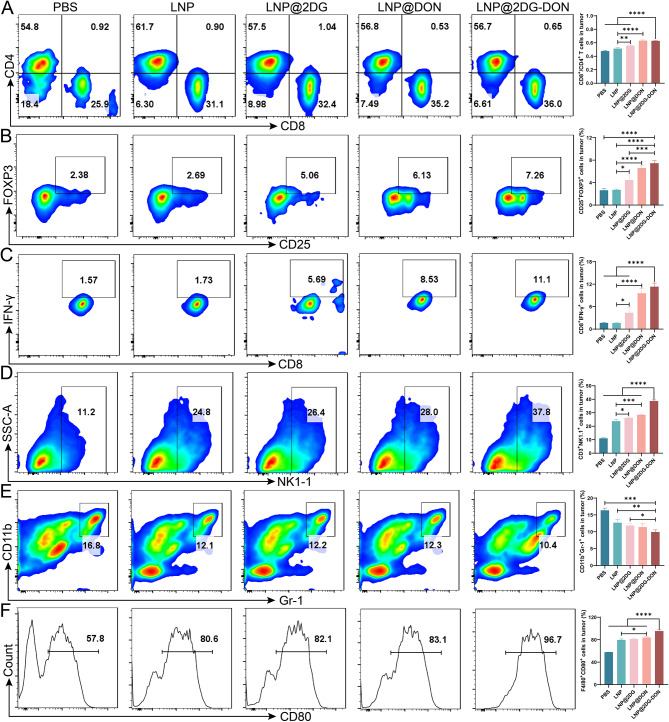
In Vivo Anti-Tumor Immune Mechanism of LNP. **A**) Tumor infiltration of CD4 + CD8+ T cells. **B**) CD25 + FOXP3+cells in the tumor tissue. **C**) CD8 + IFN-γ + cells in the tumor tissue. **D**) CD3 + NK1.1 + cells in the tumor tissue. **E**) CD11b + Gr-1 + cells in the tumor tissue. **F**) F4/80 + CD80+cells in the tumor tissue

The effect of different treatments on immune cells in the tumor tissues from the mouse xenograft was analyzed using flow cytometry. The results showed no change in CD8 + CD4+ T cells (Fig. 8A, *p* > 0.05), CD25 + FOXP3+ cells (Fig. 8B, *p* > 0.05), CD8 + IFNG+ cells (Fig. 8C, *p* > 0.05), CD3 + NK1.1+ (Fig. 8D, *p* > 0.05), CD11b + Gr-1 + cells (Fig. 8E, *p* > 0.05) and F4/80 + CD80+ cells (Fig. 8F, *p* > 0.05) in the tumor tissues between the LNP and PBS groups. However, there was a significant increase in CD8 + CD4+ T cells (Fig. 8A, *p* < 0.05), CD25 + FOXP3+ cells (Fig. 8B, *p* < 0.05), CD8 + IFNG+ cells (Fig. 8C, *p* < 0.05), CD3 + NK1.1+ (Fig. 8D, *p* < 0.05), and F4/80 + CD80+ cells (Fig. 8F, *p* < 0.05) and a significant decrease in CD11b + Gr-1 + cells (Fig. 8E, *p* < 0.05) in the tumor tissues of the LNP-loaded nano-drugs in contrast to the LNP and PBS groups. Additionally, there was a significant difference between the LNP@2DG-DON group and the LNP@DON and LNP@2DG groups (Fig. 8, *p* < 0.05).

Flow cytometry was also used to detect immune cells in the spleen of mice harboring the xenograft. The results showed that the numbers of CD8 + CD4+ cells and CD8 + CD44+CD62L- cells in the spleen were increased in the LNP@2DG group (Fig. S4A, *p* < 0.05), as well as in the LNP@DON and LNP@2DG-DON groups, with the effect of LNP@2DG-DON being significantly greater than that of LNP@DON and LNP@2DG groups (Fig. S4A, *p* < 0.05). On the contrary, a decrease in CD11b + GR-1 + cells was recorded in the LNP-loaded nano-drugs groups in contrast to the LNP and LPS groups (Fig. S4C, *p* < 0.05). In addition, the number of CD11b + GR-1 + cells was found to be significantly decreased in the LNP@2DG-DON group in contrast to the LNP@DON and LNP@2DG groups (Fig. S4C, *p* < 0.05).

Moreover, flow cytometry analysis of immune cells in the lymph of mice with xenograft showed a significant increase in the numbers of CD8 + CD4+ T cells, CD4 + CD44+CD62L- cells, CD8 + CD44+CD62L- cells CD11c+MHCII+ cells, and CD11c+CD80 + CD86+ cells in the LNP@2DG-DON group relative to the LNP@DON and LNP@2DG groups (Fig. S5A-F, *p* < 0.05). In addition, the number of CD11b + GR-1 + cells was found to be decreased in the LNP@2DG-DON group in contrast to the LNP@DON and LNP@2DG groups (Fig. S5D, *p* < 0.05). There were no significant discrepancies in the numbers of cells among the LNP group and the PBS group (Fig. S5E, *p* > 0.05).

## Discussion

Central cellular metabolism and tumor immune microenvironment play vital roles in tumorigenesis, especially in pancreatic cancer. Developing drug-delivery systems targeting these processes may be key to improving cancer treatment. In the present study, we successfully developed liposome-based nano-drugs and investigated their potential to inhibit pancreatic cancer and modulate the immune system. The results indicated that LNP-loaded 2DG, LNP-loaded DON, or dual loading of both significantly decreased the proliferation, invasion, and migration of pancreatic cancer cells while promoting their apoptosis. In vivo, we also found that these nanodrugs stimulated immune cells, including CD8 + CD4+ T cells, CD25 + FOXP3+ cells, CD8 + IFNG+ cells, CD3 + NK1.1 + cells, and F4/80 + CD80+ cells, but inhibited CD11b + GR-1 + cells in pancreatic tumors, indicating their potential as immunotherapeutics. In addition, the combined loading of 2DG and DON was more effective than single loading, positioning LNP@2DG-DON as a new candidate drug for cancer treatment, especially pancreatic cancer.

In cancer therapy, it is crucial to develop a cancer cell-specific drug carrier that minimizes non-specific drug uptake by normal cells, thus reducing severe side effects. For example, studies have shown that functionalized MSN nanoparticles, especially when engineered to include defects, have substantial potential for drug delivery, improving drug loading and release, maintaining biodegradability, and reducing systemic toxicity [41]. Functionalized systems of targeted drug delivery, including targeting ligand-functionalized systems, are gaining increased importance in making cancer therapies more specific and effective. For example, folic acid-functionalized nanodevices have been developed to release therapeutic agents in response to the acidic pH of the tumor microenvironment, thereby boosting drug delivery to the tumor site [42]. In the same way, our LNP@2DG-DON liposomal nanoparticles will target the tumor microenvironment, releasing drugs selectively to augment the therapeutic effects of the glycolysis inhibitor 2DG and the GFAT1 inhibitor DON. Additional strategies involve modulating the tumor microenvironment to improve treatment by developing siRNA nanoparticles targeting PD-L1, not only to minimize side effects but also to activate tumor immunity [43]. Due to their unique properties, LNPs have emerged as a promising drug delivery system for cancer treatment. In fact, LNPs have been used in recent studies to deliver drugs with high precision and to enable customized cancer treatment. For example, in ovarian cancer, antitumor activity of LNP-loaded cisplatin targeting CD24-positive cells has been reported [44]. In the present study, we also demonstrated the potential of LNP for the targeted delivery of 2DG and DON in pancreatic cancer therapy. Our LNP-loaded nanodrugs were spherical and exhibited a uniform size distribution, with a peak at around 120 nm. Their PDI values were low, and they had negative zeta potential, indicating their stability. These nanoparticles were efficiently taken up by pancreatic cancer cells, making them a promising candidate for drug delivery. The high LE and the efficient drug release demonstrated the effectiveness of these nanoparticles in delivering drugs to cancer cells. This could potentially lead to a reduction in the required drug dosage and minimize side effects. This is an important finding because it suggests that these nanoparticles could be used to boost the response of the immune system to cancer. In addition, the nanoparticles inhibited cancer cell proliferation and metastasis, which is significant because cancer metastasis is a major cause of cancer-related deaths and could potentially improve the prognosis of cancer patients. Thus, these results indicated that the LNPs have great potential as a drug delivery system for cancer treatment. They not only have excellent drug-delivery capabilities but can also effectively target cancer cells and boost the ability of the immune system to fight cancer.

The relationship between glucose metabolism and tumor microenvironment is necessary for cancer progression. Altering glucose metabolism can affect tumor growth, as demonstrated in previous studies [45–48]. For example, it has been previously shown that MSNs loaded with bortezomib selectively affect metabolism and cause cell death in multiple myeloma cells [49]. Here, our findings demonstrated that LNP@2DG nanoparticles could regulate cellular metabolism and affect the expression of some apoptosis-associated proteins, such as Bax, cleaved caspase 3, and Bcl2, thereby hindering the proliferation and metastasis of pancreatic cancer cells. Additionally, in mice with xenografted tumors, LNP@2DG also boosted anti-tumor immunity and inhibited tumor growth. These effects were attributed to 2DG, consistent with earlier studies that have extensively examined its role in cancer metabolism and immunotherapy. It had been demonstrated in previous research that 2DG alone [50, 51] or in combination with other treatments such as localized radioiodine therapy [52], chemoradiotherapy [53], or penfluridol [54] could potentially inhibit various cancer cell growth by targeting glycolysis. Moreover, studies have revealed that 2DG, when used together with highly resistant cancer cell viability inhibitors like metformin [55–58], polydatin [59], and buformin [55], induces the detachment of viable cancer cells.

Current research has demonstrated the potential of targeting TMUB1 in regulating PD-L1 glycosylation and polyubiquitination, thereby improving antitumor immunity [60]. Another study has highlighted the role of GFAT1, a key enzyme in the hexosamine biosynthetic pathway, in PD-L1 stability and glycosylation [13]. Hindering GFAT1 activity decreases IFNγ-induced PD-L1 levels in many lung cancer cell lines and impairs PD-L1 glycosylation, promoting its proteasomal degradation [13]. Furthermore, inhibition of GFAT1 has been shown to enhance T-cell activation and NK cell cancer-killing activity under IFNγ treatment, making it a promising candidate to improve the efficacy of lung cancer immunotherapy [13]. Herein, we report that the GFAT1 inhibitor, DON, when loaded onto LNP, exerts anticancer activity by modulating the immune response and regulating cancer cell metabolism. In addition, the combined loading of DON and 2DG on LNP had an improved inhibitory effect on PD-L1, indicating that the LNP@2DG-DON nanoparticles exert their effect by interfering with the glycosylation status of PD-L1. The 2DG/DON dual loading combines inhibitory 2DG with activating DON to target distinct but complementary metabolic and immune pathways, thereby enhancing tumor cell death and immune responses [17, 58, 61–63]. This metabolic stress, in turn, is a precondition for further therapeutic intervention in cancer cells. Inhibition of GFAT1 by DON interferes with the glycosylation of major immune checkpoints, such as PD-L1, and activates immune cells, thereby increasing anti-tumor immunity [13, 64]. These drugs exert a synergistic effect, harnessing tumor cell metabolic vulnerabilities and enhancing the immune response. These synergistic interactions align with previous findings indicating that combined treatment targeting glycolysis and immune checkpoints improves efficiency across a variety of cancers by activating PD-L1 degradation. The synergistic interactions between 2DG and DON are consistent with prior findings demonstrating the increased efficacy of combination therapy targeting glycolysis and immune checkpoints in a variety of cancers [58, 65, 66]. Our findings, thus, prove the notion that such a combination therapy would be especially effective in the treatment of pancreatic cancer, a malignancy that is notoriously difficult to treat using conventional therapies. This increased treatment effect may be explained by the dual targets of metabolic reprogramming and immune modulation, both of which are important cancer hallmarks. In addition, DON also disrupts N-glycosylation, which has been reported to mediate tumor progression and immune evasion by inhibiting GFAT1 [67, 68]. A combination of 2DG and DON makes tumor cells more susceptible to immune-mediated killing and less immunosuppressive, offering a novel, broad-range method of cancer therapy. To sum up, the synergistic interaction between 2DG and DON suggests a combination therapy focused on tumor metabolism and immune modulation, a promising approach to address the limitations of monotherapies and enhance treatment outcomes in pancreatic cancer and other malignancies.

## Supplementary Information

Below is the link to the electronic supplementary material.

Supplementary Material 1

Supplementary Material 2

## Data Availability

The datasets generated during and/or analyzed during the current study are available from the corresponding author upon reasonable request.
