# Supplementary material for: Efficacy of LNP@2DG-DON liposomal nanoparticles in tumor inhibition and immune activation
Source: J Transl Med. 2026 May 5;24:639. doi: 10.1186/s12967-026-08039-8 (PMC13151400; doi:10.1186/s12967-026-08039-8)
Supplement: Supplementary file 1 — Supplementary Material 1 [file 12967_2026_8039_MOESM1_ESM.docx]

**Supplementary Materials**

**
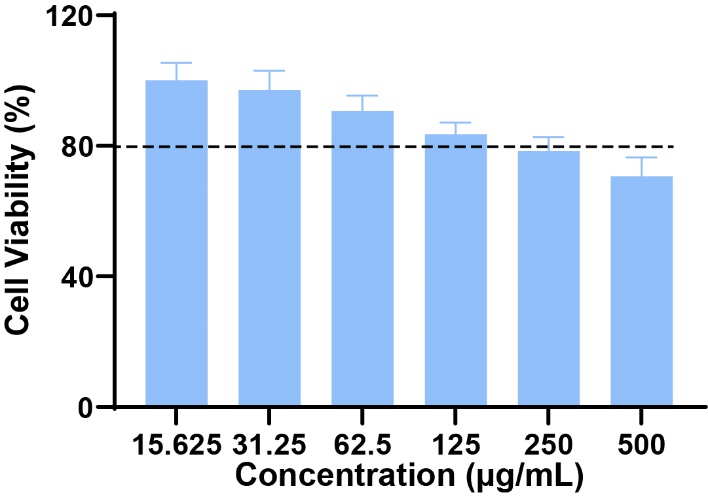
**

**Fig S1 Infrared spectra of nanoparticles.**

**
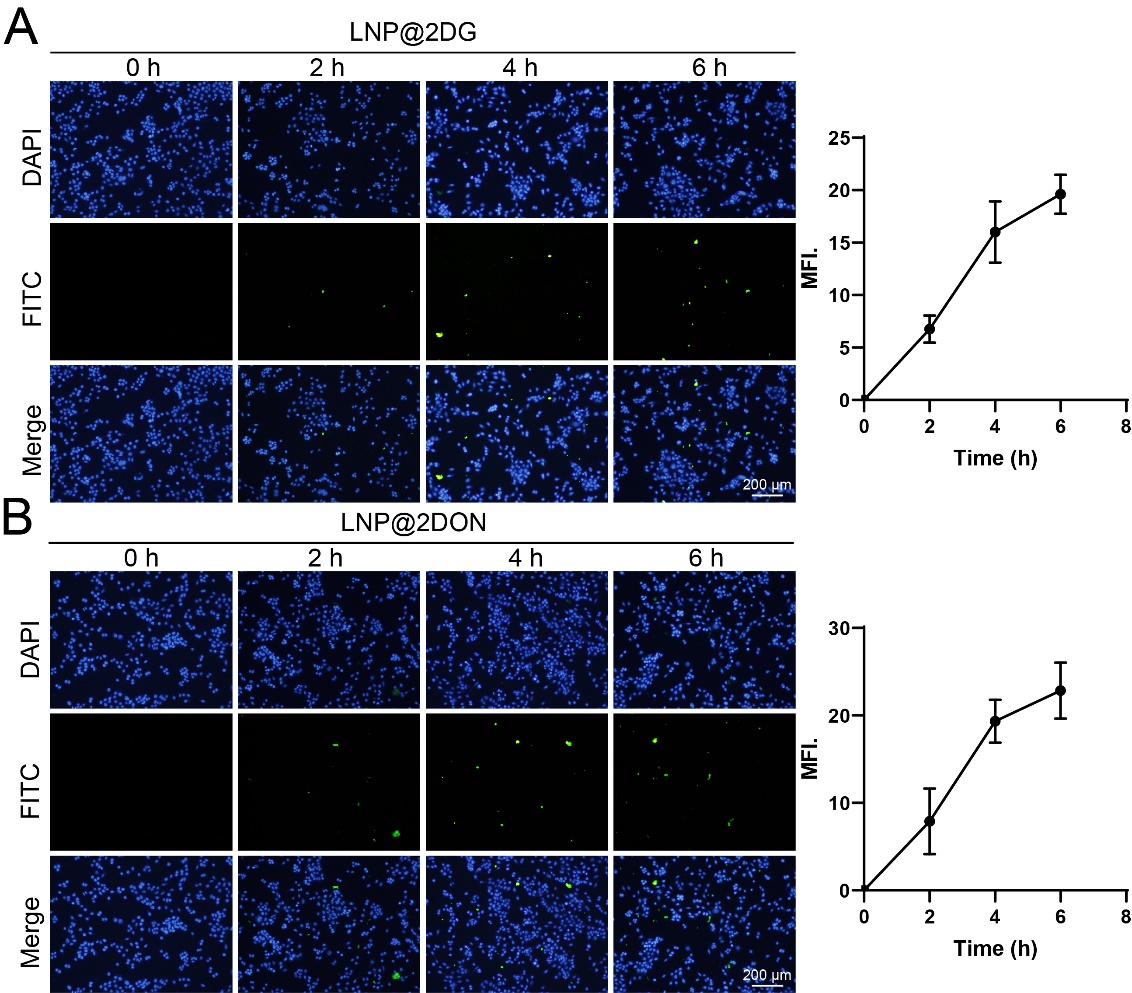
**

**Fig S2 Time changes of FITC immunofluorescence staining of ASPC-1 cells.** A)

Time-dependent uptake of FITC-labeled LNP@2DG by ASPC-1 cells, observed over 0, 2, 4, 6, 8 hours. B) Time-dependent uptake of FITC-labeled LNP@DON by ASPC-1 cells, observed over 0, 2, 4, 6, 8 hours.

**
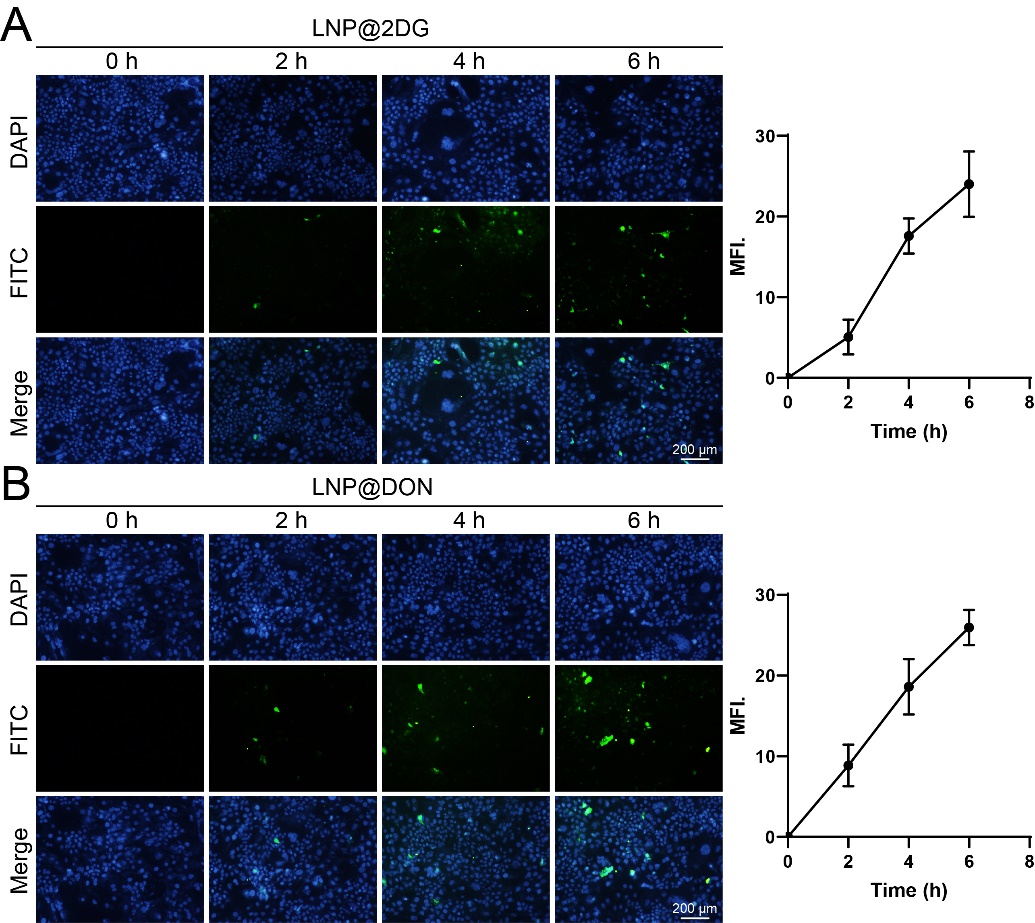
Fig S3 Time changes of FITC immunofluorescence staining of PANC-1 cells.** A)

Time-dependent uptake of FITC-labeled LNP@2DG by PANC-1 cells, observed over 0, 2, 4, 6, 8 hours. B) Time-dependent uptake of FITC-labeled LNP@DON by PANC-1 cells, observed over 0, 2, 4, 6, 8 hours.

**
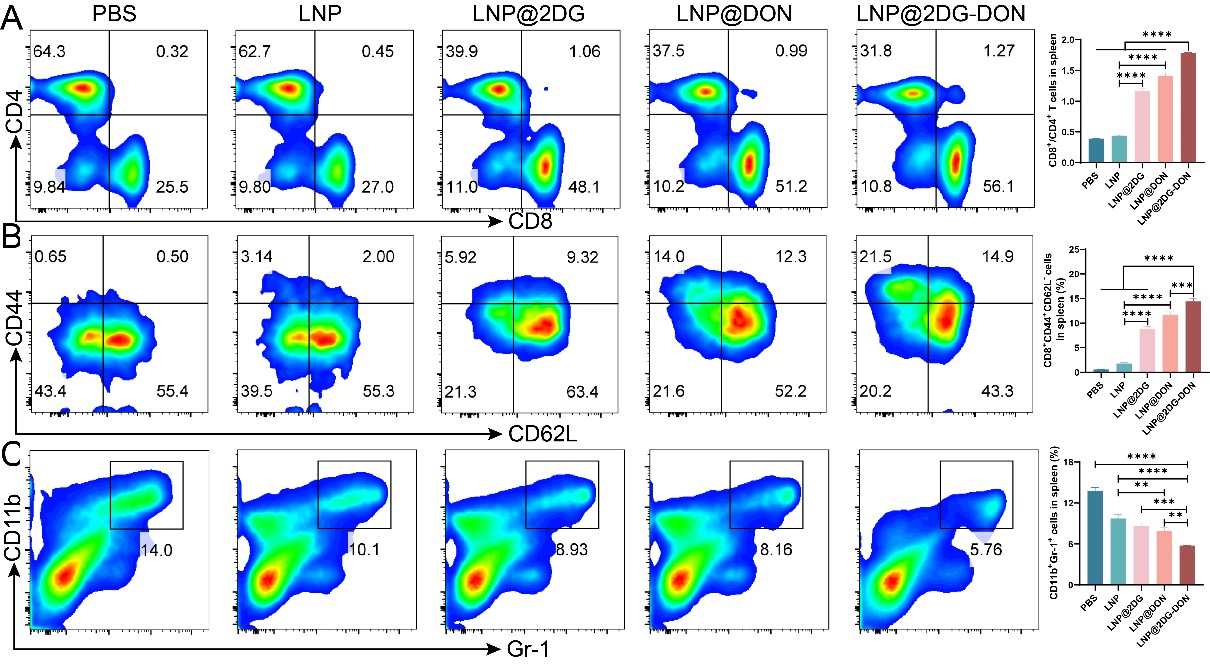
**

**Fig. S4 In Vitro Anti-Tumor Immune Mechanism of LNP.** A) Tumor infiltration of CD4+CD8+ T cells. B) CD8+CD44+CD62L+cells in the tumor tissue. C) CD11b+Gr-1+ cells in the tumor tissue.


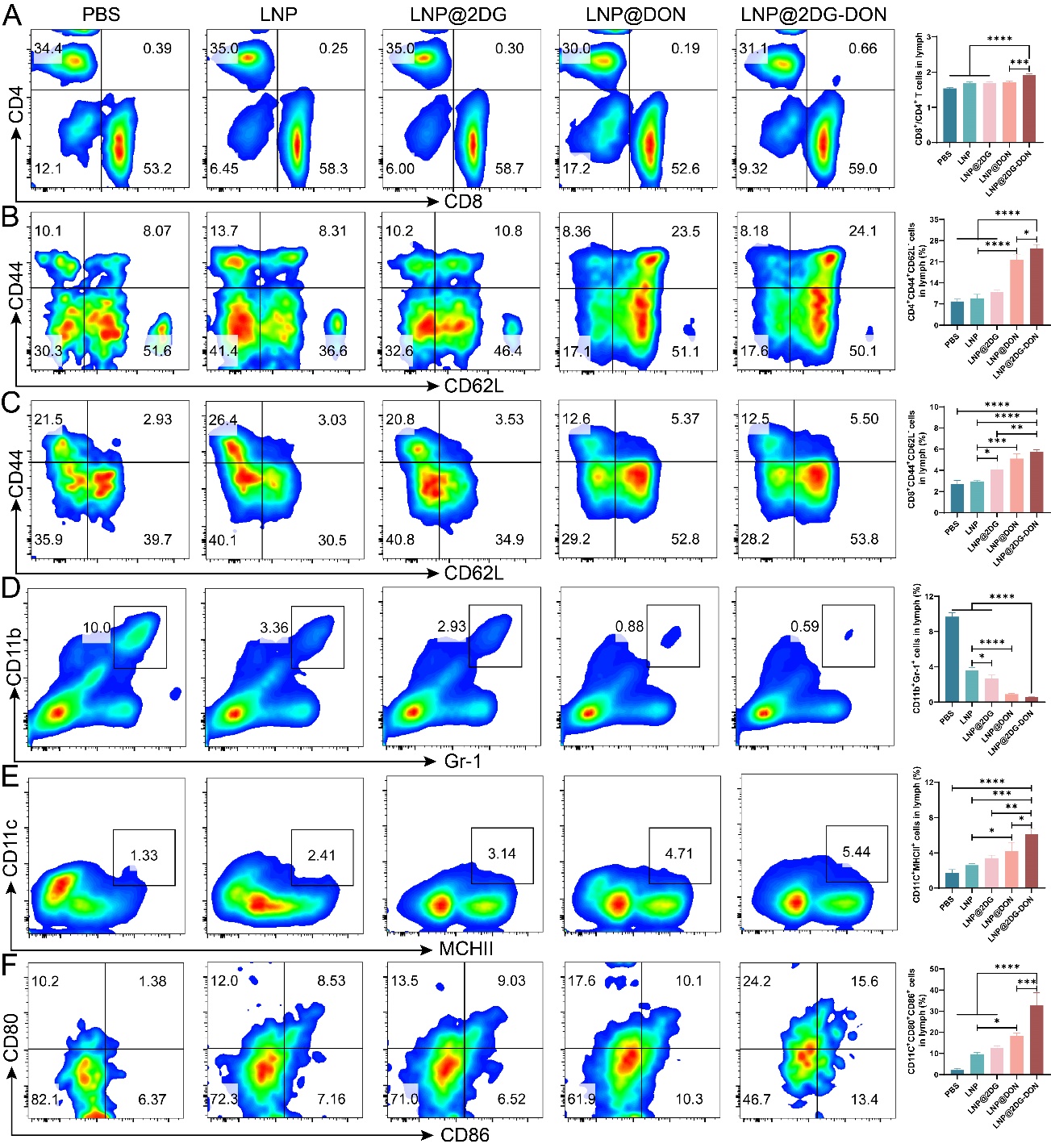
**Fig. S5 Flow cytometry analysis of immune cells in the lymph of mice.** A) Tumor infiltration of CD8+CD4+T cells. B) CD4+ CD44+CD62L+ cells in the tumor tissue. C) CD8+CD44+ CD62L+ cells in the tumor tissue. D) CD11b+ Gr-1+ cells in the tumor tissue. E) CD11c+ MCHII+ cells in the tumor tissue. F) CD80+ CD86+ cells in the tumor tissue.

**
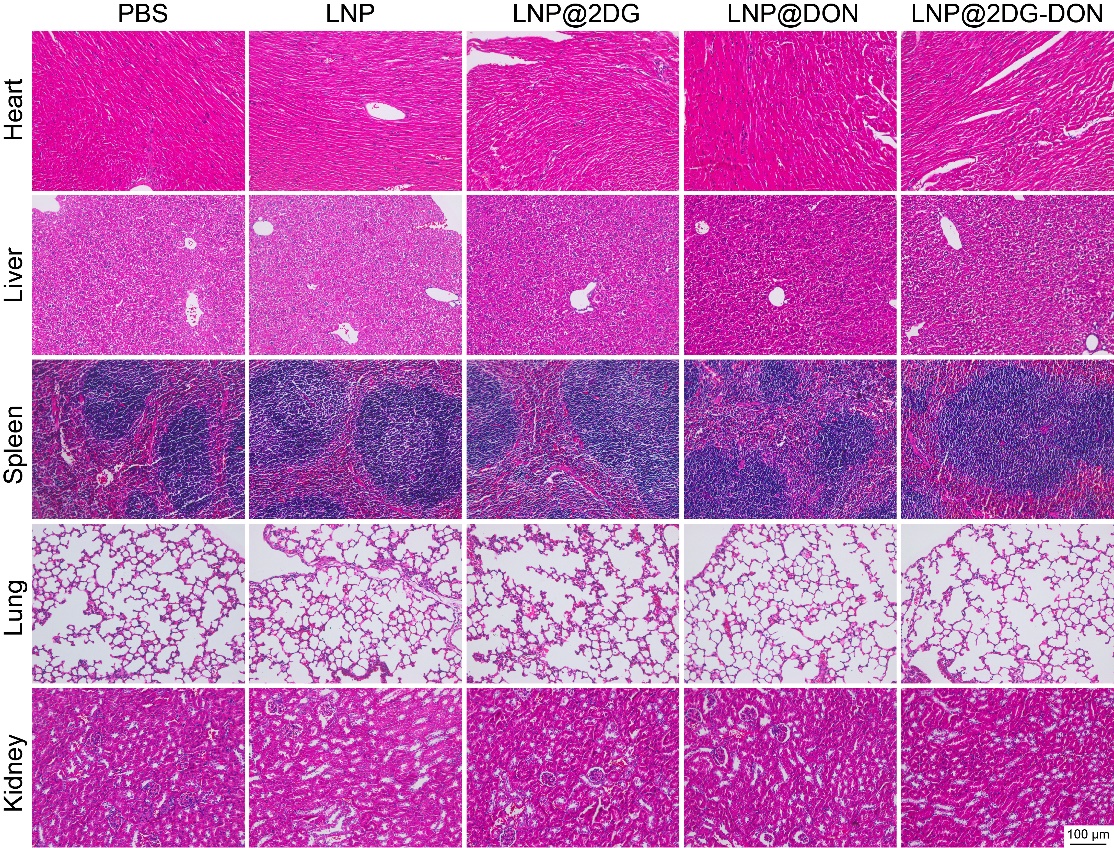
Fig. S6 H&E staining of heart, liver, spleen, lung, and kidney.**
